# Compliance With Referral Advice After Treatment With Prereferral Rectal Artesunate: A Study in 3 Sub-Saharan African Countries

**DOI:** 10.1093/cid/ciw627

**Published:** 2016-12-06

**Authors:** Mohamadou Siribié, IkeOluwapo O. Ajayi, Jesca Nsungwa-Sabiiti, Armande K. Sanou, Ayodele S. Jegede, Chinenye Afonne, Catherine O. Falade, Melba Gomes

**Affiliations:** 1 Groupe de Recherche Action en Santé, Ouagadougou, Burkina Faso; 2 Department of Epidemiology and Medical Statistics, College of Medicine; 3 Department of Sociology, Faculty of Social Sciences; 4 Epidemiology and Biostatistics Research Unit, Institute of Advanced Medical Research and Training; 5 Department of Pharmacology and Therapeutics, College of Medicine, University of Ibadan, Nigeria; 6 Child Health Division, Ministry of Health, Kampala, Uganda; 7 UNICEF/UNDP/World Bank/WHO/Special Programme for Research & Training in Tropical Diseases, World Health Organization, Geneva, Switzerland

**Keywords:** malaria, community health worker, rectal artesunate, rapid diagnostic tests, compliance with referral advice

## Abstract

***Background.*** Children aged <5 years were enrolled in a large study in 3 countries of sub-Saharan Africa because they had danger signs preventing them from being able to take oral medications. We examined compliance and factors associated with compliance with referral advice for those who were treated with rectal artesunate.

***Methods.*** Patient demographic data, speed of accessing treatment after danger signs were recognized, clinical symptoms, malaria microscopy, treatment-seeking behavior, and compliance with referral advice were obtained from case record forms of 179 children treated with prereferral rectal artesunate in a multicountry study. We held focus group discussions and key informant interviews with parents, community health workers (CHWs), and facility staff to understand the factors that deterred or facilitated compliance with referral advice.

***Results.*** There was a very high level of compliance (90%) among patients treated with prereferral rectal artesunate. Age, symptoms at baseline (prostration, impaired consciousness, convulsions, coma), and malaria status were not related to referral compliance in the analysis.

***Conclusions.*** Teaching CHWs to diagnose and treat young children with prereferral rectal artesunate is feasible in remote communities of Africa, and high compliance with referral advice can be achieved.

Most of the annual estimated malaria deaths are in young children <5 years of age, who live in areas of intense malaria transmission of *Plasmodium falciparum*, especially in sub-Saharan Africa. For many of these children, it will be difficult to distinguish clinically between malaria and pneumonia [1] and febrile sick children with positive blood smears may be initially diagnosed as having severe malaria, but the specificity of the diagnosis is likely to be poor during the malaria season when a high proportion of children are parasitemic [2]. Sepsis is underdiagnosed and difficult to differentiate from severe malaria [3, 4]. Children with *P. falciparum* parasitemia who are prostrate but fully conscious, with impaired consciousness or in coma, or who require parenteral treatment because of persistent vomiting even if they lack any specific clinical or laboratory features of severe malaria, should be treated as an emergency, as their risk of death, even with treatment, is high [5].

The progression from uncomplicated to severe malaria and death is rapid, especially in young children. The risk of death from severe malaria is highest within the first 24 hours because the lethal pathological processes in *falciparum* malaria that kill may be too advanced, but the risks can be reduced substantially by immediate and effective parenteral antimalarial treatment and specialized facility care [6–8]. However, in remote areas, caregivers may be reluctant to take their children to a facility, and for those who attempt the journey, the transit time between referral and arrival at a health facility where intravenous treatment can be given may be so long as to delay the start of treatment, leading to a poor outcome. The child can die *en route*, or upon admission [6]. It is therefore recommended that patients, particularly young children unable to take oral medication, be treated with a single dose of rectal artesunate as prereferral treatment to reduce the risk of death or permanent disability [9, 10]. When rectal artesunate is used, the patient should be transported to a facility where intramuscular or intravenous treatment is available. This strategy heavily depends upon caregivers' compliance with advice to proceed to a health facility to complete the treatment and manage nonmalarial conditions.

Other studies on compliance with prereferral treatment with rectal artesunate have shown low noncompliance ranging between 5 and 12% for those who survive at least 6 hours [10, 11]. However, data are very limited on routine compliance with referral advice and factors that may influence it. A multicenter study was conducted in Burkina Faso, Nigeria, and Uganda in children aged <5 years, providing patients diagnosed positive via a rapid diagnostic test (RDT) with Artemether-lumafantrine (Coartem®) if they could take oral drugs, and with rectal artesunate if they could not. The latter group was referred for further management to the nearest health facility. Compliance with prereferral advice and factors influencing compliance are reported here.

## METHODS

### Study Settings

The study was part of an intervention assessing the feasibility of delivering a package of malaria diagnosis and treatment in rural areas in 3 countries with different malaria epidemiology. In the component to understand compliance, quantitative and qualitative data collection techniques were used.

In Burkina Faso, the study took place in the rural health area of Sidéradougou in the southwestern part of the country, which consists of 21 administrative villages and 34 farming hamlets with a total population of about 42 000 inhabitants, including approximately 7500 children aged <5 years. Transmission of malaria is intense during the rainy season (May–October). *Plasmodium falciparum* is the main parasite species and malaria is the leading cause of consultation and hospitalization in children aged <5 years. The first level of care is the health center “Centre de Santé et de promotion sociale” managed by a nurse, and patients are referred from there to the “Centre Medicale,” which is managed by a medical doctor, and from there to the regional hospital. Both the Centre de Santé and the Centre Medicale are relatively distant from Sidéradougou, and patients seek alternative care from traditional healers, faith homes, drug shops/drug hawkers, dispensaries, and community health workers (CHWs) before going to the Centre de Santé, especially as care is fee-based. A consultation requires approximately 200 West African CFA francs (XOF) (US Dollars [USD] $.30) and admitted patients pay for their bed.

In Nigeria, in the 33 study villages, similar patterns of care are found—use of traditional healers, traditional birth attendants, CHWs, health centers, maternity wards, and private clinics (usually managed by a nurse with admission no longer than 24 hours), as well as shops/patent medicine sellers/drug hawkers where they buy chloroquine, antibiotics, and antimalarials without prescription. There are 8 health centers managed by community extension workers. The official policy is that consultations, drugs, and bed costs at public facilities are provided without charge for young children <5 years of age. However, practice is often different from policy.

Uganda has a similar variety of traditional healers, mission facilities, drug shops, and private clinics and several grades of health centers managed by different cadre of personnel. A grade II facility is an outpatient dispensary managed by a nurse/clinical officer, grade III facilities have inpatient beds and a medical doctor twice weekly in attendance, and grade IV facilities also have inpatient beds but a resident medical doctor. The 84 study villages in Uganda benefited from 2 grade IV facilities, one in each district, but no district hospital. Clinical care is provided without charge, but patients pay for hospital registration and medications.

### Procedures

During the study, children aged 6–59 months with fever or history of fever, residing and intending to reside within the community for the following 6 months, were treated with rectal artesunate if they had at least 1 danger sign. Danger signs were defined in the framework of this study for rectal artesunate as being too weak to sit, stand, or walk; unable to eat, drink, or suck; repeated vomiting; repeated convulsions; altered consciousness; or coma. Written or witnessed verbal informed consent was obtained from the guardian of each child before enrollment. Children were treated regardless of the malaria RDT result and referred. The child was not treated if there was any prior history of allergy to artemisinins or if there was prior treatment with rectal artesunate in the previous 24 hours.

After treatment, the parents/caregivers were advised to go immediately to the nearest health facility and were provided with a referral card to facilitate screening and review at the facility. A case report form was completed for each child who arrived at a health facility to document the signs and symptoms observed on arrival and the results of any diagnostic or laboratory investigations and treatments given, and to record the status on discharge. Post-treatment, 2 visits were performed by the CHW at the caregiver's home; the first visit was within 6 days after treatment and a second visit occurred within the following 7–30 days to check the health status of the child and to re-refer the child to the health facility for further examination if the child's clinical condition was suboptimal.

We interviewed parent/guardians of children who had been treated with rectal artesunate and did not comply with referral advice to proceed to the health facility after treatment. The purpose was to understand reasons for noncompliance with referral advice before the study concluded, while guardians remembered why they had not complied with referral advice. We also had focus group discussions (FGDs), key informant interviews (KIIs), and discussed case studies with caregivers. The FGDs were held separately with male and female caregivers and included guardians who had complied with referral advice and some who had not. Interviews were conducted in quiet places chosen with study participants. The study purpose was fully explained, and the discussions lasted up to 90 minutes and ended when all issues had been exhausted. All discussions were moderated by one of the study investigators assisted by a research assistant taking notes. One FGD was conducted each day to be able to reflect and consolidate the issues emerging for further questioning. In addition, there were KIIs with 5 randomly chosen CHWs who had treated with rectal artesunate and referred sick children and with 2 health facility workers. FGDs and KIIs were carried out in local languages or in French/English, based on participant preferences.

### Data Collection and Handling

Quantitative data on treatment and referral were derived from case report forms that were part of the main intervention [12], checked for inconsistencies. All FGDs and KIIs were audio-recorded, and transcribed by an experienced local transcriber who listened to the recordings in the local language and directly translated into French/English narratives. The FGDs and KIIs facilitators verified transcriptions.

### Statistical Analysis

Quantitative data were double-entered using EpiData software. Data analysis was done using Stata software, version 14. Bivariate analysis was used to assess associations between the independent variables (potential factors) and the outcome variable (compliance with referral advice). *P* = .05 was adopted as the threshold of significance. Thematic (FGD/KII) and narrative (case study) analyses of data were categorized and analyzed according to study themes. The qualitative narratives were reviewed by anthropologists without specialized software in Burkina and Uganda and using Nvivo 8 software in Nigeria. Data were anonymized. The findings generated were integrated and triangulated on presentation.

### Ethical Considerations

The research protocol of the main study was approved by the National Health Research Committee and Oyo State Ministry of Health in Nigeria; the National Ethics Committee for the Research on Health in Burkina Faso; the National Council for Science and Technology in Uganda; and the World Health Organization Research Ethics Review Committee.

## RESULTS

### Quantitative Results

Table 1 provides an overview of the 179 patients treated with rectal artesunate. Treated patients were relatively evenly distributed by gender. Most were aged <3 years, with the highest proportion in the lowest age category being in Burkina Faso. The mean age by country reflects this distribution: 21.2 months in Burkina Faso, 28.4 months in Uganda, and 29.3 months in Nigeria.

**Table 1.** Overview of Patients Receiving Rectal Artesunate Treatment

Rectal Artesunate Treatment	Burkina Faso	Nigeria	Uganda	Total
Total No. treated	139	25	15	179
Gender				
Male	78 (56.1)	9 (36.0)	0^a^	87 (48.6)^b^
Female	61 (43.9)	16 (64.0)	0^a^	77 (43.0)^b^
Child's age, mo				
<6	2 (1.4)	0 (0.0)	1 (6.7)	3 (1.7)
6–11	30 (21.6)	3 (12.0)	1 (6.7)	34 (19.0)
12–23	58 (41.7)	6 (24.0)	5 (33.3)	69 (38.5)
24–35	27 (19.4)	7 (28.0)	2 (13.3)	36 (20.1)
36–47	13 (9.4)	7 (28.0)	3 (20.0)	23 (12.8)
48–59	9 (6.5)	2 (8.0)	2 (13.3)	13 (7.3)
≥60	0 (0.0)	0 (0.0)	1 (6.7)	1 (0.6)
Age, y, mean (SD)	21.2 (12.7)	29.3 (13.8)	28.4 (17.8)	22.9 (13.7)
CNS involvement at baseline				
Yes	35 (25.2)	22 (88.0)	10 (66.7)	67 (37.4)
No	104 (74.8)	3 (12.0)	5 (33.3)	112 (62.6)
Symptoms at baseline (multiple symptoms possible)				
Cannot eat, drink, or suck	69 (49.6)	22 (88.0)	4 (26.7)	95 (53.1)
Repeated vomiting	86 (61.9)	3 (12.0)	2 (13.3)	91 (50.8)
Too weak to sit, stand, or walk	65 (46.8)	21 (84.0)	1 (6.7)	87 (48.6)
Repeated convulsions	14 (10.1)	19 (76.0)	10 (66.7)	43 (24.0)
Altered consciousness/coma	24 (17.3)	10 (40.0)	…	34 (19.0)
Time between danger signs and treatment				
Up to 6 h	30 (21.6)	7 (28.0)	2 (13.3)	39 (21.8)
>6 to 12 h	16 (11.5)	3 (12.0)	1 (6.7)	20 (11.2)
>12 to 18 h	15 (10.8)	2 (8.0)	0 (0.0)	17 (9.5)
>18 to 24 h	10 (7.2)	5 (20.0)	6 (40.0)	21 (11.7)
>24 h	64 (46.0)	8 (32.0)	1 (6.7)	73 (40.8)
Time unknown	4 (2.9)	0 (0.0)	5 (33.3)	9 (5.0)
Time between danger signs and rectal artesunate Rx, mean (SD)	27.8 (24.4)	26.4 (30.7)	19.6 (14.1)	27.1 (24.9)
Malaria positivity				
RDT positive	82 (59.0)	25 (100.0)	13 (86.7)	120 (67.0)
RDT negative	50 (36.0)	…	2 (13.3)	52 (29.1)

Data are presented as No. (%) unless otherwise indicated.

Abbreviations: CNS, central nervous system; RDT, rapid diagnostic test; Rx, treatment; SD, standard deviation.

^a^ Gender was not documented in Uganda.

^b^ Total/Percent excludes Uganda.

Children with clear central nervous system (CNS) involvement (repeated convulsions, altered consciousness, or coma) at baseline assessment are separated from those with other symptoms preventing oral treatment (inability to eat, drink, or suck; inability to sit, stand, or walk; prostration) at baseline. Table 1 shows that the percentage of children coming for treatment with CNS malaria was much smaller in Burkina Faso (25.2%) compared with either Nigeria or Uganda, where children with CNS symptoms were 66.7% in Uganda and 88% in Nigeria. Specificity of the clinical symptoms reflect this distribution, with a greater proportion of children in Burkina Faso having non-CNS symptoms—inability to eat, drink, or suck (49.6%); too weak to sit, stand, or walk (46.8%); or with repeated vomiting (61.9%)—than the other 2 countries. In Nigeria, the majority of patients came for treatment with repeated convulsions or altered consciousness/coma, whereas in Uganda the majority of patients came because of repeated convulsions.

The delay between danger signs being observed by parents and arrival for treatment shows that in Burkina Faso, half of the children (51%) arrived within 24 hours of first symptoms, whereas in general a nonsignificant shorter time to arrival was noted in Nigeria and Uganda: 27.8 hours in Burkina vs 26.4 hours in Nigeria and 19.6 hours in Uganda. However, an important proportion of patients delayed treatment even when their children had danger signs—from 46% in Burkina Faso, 32% in Nigeria, and 6.7% in Uganda.

Not all who met the clinical eligibility criteria for rectal artesunate were RDT positive. RDT results varied from 59% positivity in Burkina Faso, which had the largest number of patients, to 100% in Nigeria, and 86.7% in Uganda.

Table 2 provides information on whether and how the family complied with referral advice to proceed to the nearest health facility. Information documenting time of arrival was noted at hospital. Compliance with referral advice was extremely high for Burkina Faso, where 97.8% of patients arrived at the hospital, compared with 72% in Nigeria and 46.7% in Uganda. Those who complied did so mainly within the first 24 hours (90.6% in Burkina Faso and 72% in Nigeria), and the majority arrived within 6 hours post-treatment (85.9% in Burkina Faso and 88.9% in Nigeria). Symptoms at baseline did not appear to influence delays to arrival at the referral facility.

**Table 2.** Compliance With Referral Advice to Proceed to Health Facility^a^

	BURKINA FASO		NIGERIA`		UGANDA		TOTAL	
Characteristic	N	%	N	%	N	%	N	%
Overall compliance								
Yes	135	97.1	18	72.0	7	46.7	161	89.4
Compliance within 6 h^a^								
Yes	116	85.9	16	88.9	3	75.0	135	86.0
No	19	14.1	2	11.1	1	25.0	22	14.0
Time not documented	4		7		11		22	
Speed of compliance^a^								
Up to 6 h	116	85.9	16	88.9	3	75.0	135	86.0
>6–12 h	7	5.2	…	…	1	25.0	8	5.1
>12–18 h	1	0.7	2	11.1	…	…	3	1.9
>18–24 h	2	1.5	…	…	…	…	2	1.3
>24 h	9	6.7	…	…	…	…	9	5.7
Time not documented	4		7		11		22	
Where patients went post-treatment								
Health facility	136	97.8	18	72.0	7	46.7	161	89.9
Drug shop	…	…	2	8.0	1	6.7	3	1.7
Traditional healer	3	2.2	…	…	1	6.7	4	2.2
No action	…	…	2	8.0	5	33.3	7	3.9
Not Known	…	…	3	12.0	1	6.7	4	2.2
	N	Mean delay	N	Mean delay	N	Mean delay	N	Mean delay
Time from treatment to facility arrival, h, by baseline symptoms								
Cannot eat, drink or suck								
Yes	66	4.7	15	2.6	1	11.5	82	4.4
No	68	4.0	3	2.2	3	2.0	74	3.9
Repeated vomiting								
Yes	86	4.0	1	0.5	1	1	88	3.9
No	48	5.0	17	2.7	3	5.5	68	4.5
Altered consciousness/coma								
Yes	24	6.6	8	0.98	…	…	32	5.2
No	110	3.9	10	3.8	4	4.4	124	3.9
Repeated convulsions								
Yes	14	5.3	14	2.99	2	2.5	30	4.0
No	120	4.2	4	1.10	2	6.3	126	4.2

^a^ Denominators are different because data are not available for all patients.

At the first follow-up visit of each treated child, parents were asked where the child had been taken post-treatment if they did not come to the referral facility. Among the few cases in Uganda, one-third took no action after treatment, as did 8% of cases treated in Nigeria. An equal proportion of noncompliant cases went to drug shops. We have no information on compliance for 4 of the total 179 treated cases; 1 child in Nigeria died on the way to hospital.

Table 3 shows episode and patient characteristics in relation to compliance. Among the very few noncompliers with referral advice, none of the factors assessed—influenced compliance.

**Table 3.** Association Between Compliance With Referral Advice and Patient/Episode Characteristics

Characteristic	Before 6 h	After 6 h	Total	OR/RR (95% CI); *P* Value
Patients with data on time to facility	135	22	157	
Gender^a^				
Male	71 (52.6)	12 (54.5)	83	
Female	61 (45.2)	9 (40.9)	70	OR: 0.87 (95% CI, .35–2.16); .7744
Age group				
0–35 mo	107 (79.3)	19 (86.4)	126 (80.3)	
36–60 mo	28 (20.7)	3 (13.6)	31 (19.7)	OR: 0.60 (95% CI, .17–2.06); .4376
Danger signs				
1 danger sign	50 (37.0)	5 (22.7)	55 (35.0)	
>1 danger sign	85 (63.0)	17 (77.3)	102 (65.0)	OR: 2 (95% CI, .71–5.53); .1920
CNS involvement at baseline				
No	91 (67.4)	12 (54.5)	103 (65.6)	
Yes	44 (32.6)	10 (45.5)	54 (34.4)	OR: 1.72 (95% CI, .70–4.21); .2389
Access to RA within 24 h^b^				
<24 h	74 (55.6)	11 (55.0)	85 (55.6)	
≥24 h	59 (44.4)	9 (45.0)	68 (44.4)	OR: 1.02 (95% CI, .40–2.58); .9572
RDT at baseline (by CHW)^b^				
Positive	89 (69.0)	10 (47.6)	99 (66.0)	
Negative	40 (31.0)	11 (52.4)	51 (34.0)	OR: 2.45 (95% CI, .98–6.10); .0552
QC smear result^b^				
Positive	25 (32.5)	5 (33.3)	30 (32.6)	
Negative	52 (67.5)	10 (66.7)	62 (67.4)	OR: .96 (95% CI, .31–2.97); .9478

Data are presented as No. (%) unless otherwise indicated.

Abbreviations: CHW, community health worker; CI, confidence interval; CNS, central nervous system; OR, odds ratio; QC, quality control; RA, rectal artesunate; RDT, rapid diagnostic test; RR, risk ratio.

^a^ Not collected for Uganda.

^b^ Denominators differ because results are not available for all patients.

### Qualitative Data

In FGDs and KIIs carried out in Burkina Faso and Nigeria on why parents do not follow referral advice, there was agreement that integration of RDTs and prereferral rectal artesunate in the community increased access to initiation of treatment for severe malaria, but CHWs were unanimous that costs and uncertainty about facility costs were the primary deterrents in compliance with referral advice:

*When speaking about referral, some people panic in the fields  …  they can imagine only expenses.  …  others remain silent for a moment.  …  others, given the symptoms of the child  …  press you to give them a referral card.* — CHW in Burkina Faso

An example of the context in which the mother did not proceed to hospital is provided in a case treated in Nigeria. A child with febrile convulsions who had become very weak was treated with bitter leaves and palm oil rubbed over the body. When the child did not improve, the mother consulted with a CHW, who diagnosed via an RDT, treated with rectal artesunate, and advised the mother to proceed to hospital because the child had severe malaria. The mother did not go to the referral hospital and the fever continued intermittently:

*I started crying because I was scared  …  . I asked – so where will I get the money? I told her that I needed to have something in my pocket because they will ask me to buy this or pay for that.* — Case study No. 3, Nigeria

The child's condition improved after rectal artesunate treatment, and the child was later given Coartem® by the CHW to complete treatment.

In a fatal case, the need to act rapidly was emphasized. The parents of a 1-year-old child who lived 15 minutes away from the nearest hospital narrated that their child had been treated at home with paracetamol (acetaminophen) to reduce fever. At the time, the child's father was absent from home. The next day when the child did not improve, the mother went to the CHW, who treated with rectal artesunate and referred to hospital after the RDT diagnosis. The child died 30 minutes after treatment, while transport was sought to proceed to the hospital. Both parents reflected on their experience:

*It was very fast  …  when the temperature was high on the previous evening, if I had gone to the CHW, it* [*the death*] *would have been averted. I was probably not fast enough  …  *

*If one of my children is hot now, I will go quickly to the CHW without using another drug. I have learned many lessons.* — Mother and Father, Case 4, Nigeria

The rapid progression to severe malaria and death is emphasized in a 4-year-old child. The father explained that about 6 am the mother had noted the child convulsing:

*Immediately we took him to his uncle, the CHW who lives in the same village  …  He saw the child convulsing and said this was a danger sign, severe malaria  …  We were all in a state of panic  …   The CHW administered 2 rectal artesunate suppositories before advising that we should proceed immediately to the medical center so that the child obtained supplementary care  …  The previous night the child had been playing normally. We arrived around 7  …   and by 9:30 he had died.* — Father, child aged 4 years, Burkina Faso

Unanimously all guardians interviewed agreed that costs explain refusals or delays in immediate adherence to referral advice:

*When we go there* [*the hospital*] *they don't give drugs, they write the drug for us to go and buy outside  …  When we got to the hospital they checked the child and asked us to pay for a card, but we did not have any money  …  they left us outside and told us that we needed to bring money to buy blood for the child. We were outside till 10 am when the “research team” came and paid for the card and blood.* — FGD parent, Nigeria

The child was eventually treated at the hospital and transfused (cost: Nigerian naira [NGN] 500 USD $1.5 for the card, and NGN 8000 USD $25.5 for the blood transfusion), and eventually discharged.

## DISCUSSION

Overall compliance with referral advice to proceed to hospital was very high among patients treated with rectal artesunate and highest in the country with the most malaria patients [12]. The largest proportion of children completing referral arrived at the referral facility within 6 hours of being treated. This level of success with compliance is likely to be due to the level of initial and refresher training provided to CHWs together with tests and certification before their deployment. In Burkina Faso, 19 of 50 CHWs were not certified to treat with rectal artesunate, although they were used in the uncomplicated component of the study since they passed that certification. The low numbers treated with prereferral rectal artesunate in this study is likely to be due to the high numbers receiving artemisinin-based combination therapies (ACTs) on the basis of an RDT result in the different communities, thus reducing the potential to progress to severe malaria [13].

Among the relatively small proportion of patients who did not follow referral advice, we found no risk factor—gender, age, symptoms, diagnosis—that could satisfactorily explain noncompliance with referral advice. In contrast, our qualitative interviews with parents who did and did not comply with referral advice unanimously suggested that actual and anticipated cost at the referral facility was the primary constraint to proceeding with referral advice. Financial constraints were observed to delay arrival at the facility because parents had to find the means to pay for transport costs; even when referral advice was followed, the case studies in Nigeria demonstrate that some patients with severe malaria were not admitted on arrival until they could demonstrate ability to pay for admission and case management there, only made possible by the research team.

There was a wide range of symptoms among children treated. In Burkina Faso, 75% of the children were prostrated but had no symptoms of cerebral involvement (such as repeated convulsions, altered consciousness, or coma), suggesting that they came earlier for treatment than children in Nigeria and Uganda. The broader range of symptoms describing prostration and preventing intake of oral medicines has been noted in other community-based studies of rectal artesunate [10].

The qualitative and quantitative data indicate that the intervention was well accepted in the community. The high level of compliance with referral advice, and the successful integration of RDT, ACT, and prereferral rectal artesunate at the community level indicate that this package has the potential to improve access to services, particularly for patients who are at highest risk of death.

An important finding (elucidated both quantitatively and in the narratives) relates to delays in presenting for treatment among those who were neither distant from a hospital or the CHW. A significant proportion of patients delayed treatment >24 hours after initial symptoms before presenting to the CHW for prereferral rectal artesunate treatment. A few children who delayed subsequently reported taking no further post-treatment action because the child had improved and could move on to consolidation treatment. However, some children came too late for treatment and died *before* they could proceed to hospital. Thus, they came for treatment after the pathological processes had advanced beyond the ability of this drug to provide benefit. In routine deployment, presenting before CNS symptoms needs emphasis: treatment benefits patients who come early.

## Supplementary Data

Supplementary materials are available at http://cid.oxfordjournals.org. Consisting of data provided by the author to benefit the reader, the posted materials are not copyedited and are the sole responsibility of the author, so questions or comments should be addressed to the author.
